# Hidden Workers in Aging Australia: Protocol of Intersectionality-Informed Mixed Methods Study

**DOI:** 10.2196/83401

**Published:** 2025-12-31

**Authors:** Sora Lee, Woojin Kang, Lu Yang, Mehak Batra

**Affiliations:** 1 Department of Public Health School of Psychology and Public Health La Trobe Univeristy Melbourne Australia; 2 Department of Economics Hanbat National University Daejeon Republic of Korea

**Keywords:** employment, unemployment, aging workers, aging workers, hidden workers, intersectionality, health status disparities, mixed methods

## Abstract

**Background:**

Australians are living longer and are expected to remain in the workforce for longer; yet, many older adults struggle to secure employment despite being willing and able to work. A growing share of these individuals are “hidden workers,” those underused in the labor market due to missed hours, long-term unemployment, or withdrawal from job seeking despite the capacity to work. This group reflects a global trend of aging yet underused workforces, and in Australia, they represent a significant proportion of the working-age population. Addressing the challenges of hidden workers is crucial, as their inclusion could help meet labor market demands, alleviate fiscal pressures of aging, and promote healthier, more equitable aging trajectories.

**Objective:**

This intersectional mixed methods study has 3 overarching aims. First, to investigate how intersecting social identities (eg, age, gender, cultural background, health status, and caregiving responsibilities) shape hidden workforce participation and associated health outcomes among aging Australians. Second, to compare hidden workers with currently employed populations in order to identify health discrepancies between the 2 groups. Third, to explore the lived experiences of hidden workers, focusing on how intersecting and multiply disadvantaged identities impose additional burdens on employment outcomes and health status. Together, these aims will generate an integrated understanding of both structural and lived dimensions of hidden work, providing evidence to inform more equitable labor market and health policies.

**Methods:**

This study uses an explanatory sequential mixed methods design to investigate the health, resources, and employment experiences of aging hidden workers in Australia. In phase 1, an online cross-sectional survey was administered to 1166 participants (696 hidden workers aged more than 45 years and 470 current workers), capturing variables on employment history, health, discrimination, workplace social capital, caregiving, and socioeconomic status. Validated instruments, including the Workplace Age Discrimination Scale, Intersectional Anticipated Discrimination Scale, and Workplace Social Capital Index, were incorporated to ensure reliability. Phase 2 will involve semistructured interviews with a purposive subsample (30 participants) identified from survey results, focusing on lived experiences of workforce exclusion and intersecting barriers. In phase 3, quantitative and qualitative findings will be integrated through triangulation and complementarity to provide a comprehensive understanding of hidden workers’ challenges and assets, generating evidence to inform policy and stakeholder recommendations.

**Results:**

As of September 2025, the online survey has been completed, phase 2 interviews are underway, and phase 3 integration is scheduled for completion by mid-2026.

**Conclusions:**

This study will generate the first intersectional evidence on the health and employment challenges of hidden aging workers in Australia. These insights will inform tailored policy interventions that can support re-engagement, reduce inequities in health and well-being, and strengthen workforce participation. Ultimately, the findings will contribute to addressing skills shortages while promoting social and economic inclusion of aging Australians.

**International Registered Report Identifier (IRRID):**

DERR1-10.2196/83401

## Introduction

### Aging Workforce and Hidden Workers

In this study, “aging workers” refers to adults aged 45 years and older, consistent with the Department of Employment and Workplace Relations (DEWR) definition of “mature-aged” [1]. Australians are living longer and expected to work for longer than ever before. With increased life expectancy and more years of disability-free living, aging Australians have both greater capacity and greater need to remain in the workforce [2]. Many express a desire to continue working or to increase their hours; however, despite decades of experience, aging job seekers often struggle to gain employment.

Maestas and Li [3] estimated that 13% of aging workers become discouraged workers or hidden workers, meaning those not actively seeking employment despite being willing and able to work, often due to repeated rejection or perceived lack of opportunities [4]. Moreover, many discouraged workers face age-based stereotyping and discrimination. Aging “hidden workers” reflect a global trend of an aging yet underused workforce [5].

Fuller et al [6] coined the term “hidden workers” to include 3 categories of labor underuse, namely “missing hours” (working one or more part-time jobs but willing and able to work full-time), “missing from work” (unemployed for a long time but seeking employment), and “missing from the workforce” (not working and not seeking employment but willing and able to work under the right circumstances). In Australia, hidden workers comprise approximately 22% of the population aged 15 years and older [7]. However, research has devoted far less attention to their drivers and characteristics compared to the unemployed [8].

At the same time, companies are struggling to find people with skills and competencies [9]. A total of 64% of HR leaders say their companies are struggling with skills shortages; yet, only 4% of employers have any formal plan to keep aging workers in the workforce [10]. Barriers for hidden workers include skill mismatch, digital exclusion, health constraints, caregiving duties, and discriminatory hiring practices. Inflexible work arrangements exacerbate these challenges, especially for those managing care or health conditions [6]. Given these conditions, retaining and re-engaging aging workers offers an important solution to both skill shortages and the health benefits associated with meaningful work [11-13].

Therefore, better understanding and addressing the hidden workers among aging populations is important because they represent an underused economic resource that can offset the fiscal pressures of population aging [3]. At the same time, they are a systemically excluded group with notable implications for inequitable health and well-being trajectories associated with labor market discrimination [14].

### Health Inequalities

Research shows that both unemployment and underemployment harm mental and physical health. Unemployment can leave lasting “scarring” effects on individuals’ careers—including reduced wages, diminished job quality, lower aspirations, and fewer long-term achievements—even decades after the initial job loss [15]. Underemployment also affects psychological well-being, with people reporting poorer mental health and reduced life satisfaction, which can in turn impact overall health outcomes [16-18].

For aging jobseekers, the impact is amplified. According to one study [9], Australians aged 55 years and older experience median unemployment durations more than twice those of people aged 25-44 years. Prolonged unemployment erodes financial security, increases social isolation, and harms both physical and mental health, ultimately reducing re-employment prospects and life satisfaction [19].

Addressing these issues requires a systems approach that transcends individual job-search interventions. Coordinated action across employment, health, housing, and social protection systems is essential to reduce the structural barriers that sustain labor-market detachment. Age discrimination remains a major impediment to re-employment [20]. Aging interacts with race, gender, and class to shape unique “career-capital” trajectories [21-23], while organizational cultures and recruitment practices often perpetuate negative stereotypes—portraying aging workers as costly, less adaptable, or technologically resistant [24,25]. Such biases reinforce exclusion, deepen health inequalities, and constrain healthy aging.

Thus, understanding the health consequences of labor underuse among aging hidden workers—and how these are compounded by intersecting inequalities—is vital for equitable aging and workforce policy.

### Intersectional Disadvantage

Intersectionality provides a framework for understanding how multiple social identities—such as age, gender, ethnicity, health, and caregiving—combine to create unique patterns of disadvantage. Aging hidden workers are not a homogeneous group but experience interlocking systems of exclusion that shape both their employment and health trajectories [6,26].

Research highlights that aging intersects with other forms of inequality—such as race, gender, and class—to shape individualized career-capital pathways that accumulate advantage or disadvantage across the life course. For instance, women’s careers are more frequently interrupted by caregiving, leading to reduced lifetime earnings, superannuation, and access to employer-sponsored training [27-29]. Migrant and racialized aging workers often experience credential discounting and language-based discrimination, which restrict entry into professional occupations and limit career progression [30,31]. Class position also affects the capacity to invest in continuous learning, digital upskilling, and health resources that underpin employability in later life [6]. Over time, these intersecting inequalities produce differentiated career-capital trajectories. While some aging adults can leverage accumulated skills and networks to remain employable, others face compounded exclusion that intensifies their risk of becoming hidden workers [23].

ABS (Australian Bureau of Statistics) has recently published potential worker statistics that show there are 1.7 million potential workers who are willing to work but can’t [9]. Many of these individuals—particularly aging carers, migrants, or people with chronic conditions—no longer self-identify as job seekers due to discouragement, yet they remain part of the hidden workforce. Addressing this evidence gap is critical for designing equitable, targeted policy interventions.

In summary, the literature underscores that aging hidden workers represent both an underused economic resource and a systemically excluded population whose marginalization carries significant social and health costs. Addressing their exclusion is not merely a matter of fairness but also essential for maintaining workforce sustainability and reducing fiscal pressures associated with population aging. In the Australian context, meaningful progress requires a coordinated, systems-level response that goes beyond individual employability programs to integrate employment services, health care, housing, and social protection policies. However, despite clear evidence of need, little is known about the distinct circumstances of aging jobseekers, carers, and people with health conditions who have withdrawn from the labor market due to discouragement. Closing this evidence gap through intersectional and mixed methods research is therefore critical to developing targeted, evidence-based interventions that can reduce health inequities, support re-engagement, and restore opportunity structures for Australia’s aging workforce.

### Aims and Objectives

The aims and objectives are as follows:

1. Aim: to examine how intersecting social identities shape labor-market participation and health outcomes among aging hidden workers in Australia.
2. Objectives: (1) quantify disparities between hidden and active workers, (2) identify determinants of health and employment inequalities, and (3) explore lived experiences and assets among intersectionally marginalized hidden workers.

### Theoretical Framework: The Intersectionality-Based Hidden Workers’ Health Inequality Framework

The Intersectionality-based Hidden Workers Health Inequality Framework (IHHIF) guides the conceptual and analytic approach of this study. The framework integrates principles of intersectionality [27,32-34], social determinants of health [35], labor-market discrimination [6,20,23,26], and behavioral theories related to self-efficacy and aging-work motivation [36,37] to explain how employment exclusion shapes health outcomes among aging hidden workers.

IHHIF conceptualizes labor-market exclusion as the product of interacting structural and identity-based mechanisms rather than isolated individual factors. Structural determinants—such as labor policies, welfare regulations, employer practices, digital access, and socioeconomic conditions—create material and institutional constraints that influence workers’ opportunities. These structural forces intersect with social identities, including age, gender, ethnicity, migration background, class, disability, and caregiving roles, producing cumulative and patterned disadvantages over the life course.

The framework positions cognitive–behavioral mediators—such as perceived discrimination, self-efficacy, outcome expectations, and aging-related beliefs—as the mechanisms through which structural inequities become embodied and affect health, agency, and employment behaviors. These mediators are shaped not only by personal experiences but also by societal narratives about older workers, stereotypes about productivity, and the intersectional burdens of racialization, gendered care roles, or chronic illness.

IHHIF is designed to capture how hidden workers navigate the labor market.

Observable outcomes:

1. Structural determinants (policies, systems, and contexts)
2. Intersecting identities (age×gender×ethnicity×class×health status)
3. Cognitive–behavioral processes (motivation, confidence, and expectations)
4. Health and employment outcomes (physical, mental, social, and economic)
5. Assets and protective factors (social capital, community support, retraining, and resilience)

### Interact Dynamically Over Time to Shape Hidden-Worker Trajectories

By grounding this study in IHHIF, the framework enables a multilevel, intersectional, and mixed methods analysis that aligns quantitative patterns with lived experiences. It guides the identification of inequities, the interpretation of mechanisms underlying those inequities, and the development of targeted, contextually informed policy and practice recommendations to support aging hidden workers’ re-engagement and well-being.

## Methods

### Data Collection: Mixed Methods Research Process

This is a cross-sectional mixed methods approach to develop a rich understanding of study participants’ mental and physical health, coping strategies, access to resources, and informal and formal support for employment access. We will explore relevant assets and strengths for hidden workers and examine variations through the lens of intersectionality. Analyses, outputs, and dissemination plans will be planned to inform new findings to key stakeholders.

**Figure 1:**
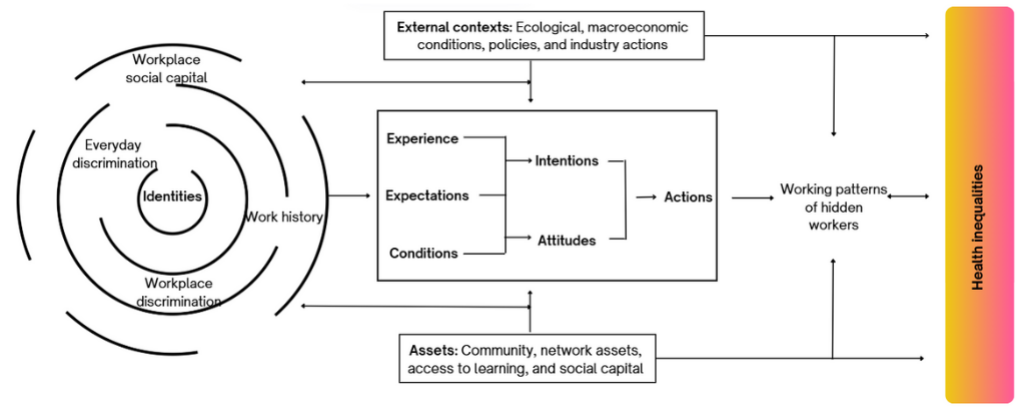
Intersectionality-Based Hidden Workers Health Inequality Framework (IHHIF).

The study is an explanatory sequential mixed methods design in which a quantitative element is used first and followed by a qualitative component contingent on and informed by the initial quantitative results. Preliminary findings from the quantitative analysis will inform later phases of qualitative data collection, and hypotheses derived from qualitative analysis will be tested in the quantitative component. A value of this design is that it enables researchers to further explore the nuances and mechanisms that explain the quantitative results [38]. The project will have 3 phases of gathering different resources. Figure 1 provides an overview of the steps taken in the data collection and analysis.

The explanatory sequential mixed methods design integrates quantitative survey data with qualitative interviews to provide a comprehensive understanding of aging hidden workers’ employment and health experiences.

### Phase 1: Online Survey

The first phase will comprise an online cross‐sectional survey administered to both hidden and nonhidden workers using the QuestionPro platform from December 2024 to March 2025. This platform provided access to a large, diverse participant pool across all Australian states and territories, enabling recruitment that captures variation in demographic, socioeconomic, and employment characteristics [39]. The survey is designed to ensure representativeness where possible and to capture key variables relevant to employment status, work history, health, and caregiving responsibilities. The full survey is included in the Multimedia Appendix 1 to ensure transparency and support replicability.

The questions will be used to (1) examine the circumstances and lived experiences for hidden workers, particularly those facing multiple and intersectional forms of marginalization (eg, cultural and linguistic diversity, disability, and agism), (2) discern the differences of attitudes, behavior, and health status between hidden and nonhidden workers, with a focus on their perspectives, challenges, and opportunities related to labor market participation. The questionnaire was pilot-tested with 30 participants to assess clarity, length, and skip-logic prior to launch. Participants were recruited through QuestionPro’s Australian panel (stratified by age, gender, and state) and included community partners (libraries and senior associations) to ensure diversity.

The total sample for the survey is 1166 participants, where 696 are selected as hidden workers aged more than 45 years, and 470 are currently in the workforce. A total sample of 1166 affords ~±4.4% precision for group proportions, detects 6-7 pp differences in prevalence at 80% power, supports multivariable regression with adequate events per variable, and provides sufficient stability for latent class analysis–based subgroup identification and subsequent policy targeting—meeting both statistical power and policy-interpretability needs.

The decision to have 45 years as our age cutoff is based on the Australian context. While there exist discrepancies between definitions of “mature aged” across different countries and various organizations, the DEWR defines ‘mature age’ to include persons aged more than 45 years [1]. Including 45- to 54-year-olds captures midlife transitions preceding traditional “older” thresholds, aligning with mature-age category and international definitions used by ILO (International Labour Organization) and OECD (Organisation for Economic Co-operation and Development [1]). This will be the first attempt to use the survey to ask 2 groups (currently working and hidden workers), enabling us to discern, for the first time, the differences in work expectations and perceptions toward their later working lives. This would allow the researcher to identify the composition of groups with single and multiple social disadvantages, which in turn would inform the population groups for the in-depth interview (phase 2). These questions are multiple-choice, simple-answer, and scale questions. The survey is anonymous, and participants can leave the survey anytime. It is expected to take approximately 15-20 minutes to complete.

Various measures of the discrimination index will be collected. The 45 years and older are exposed to accumulated lifelong discrimination and inequality. Until recently, few studies had explicitly investigated discrimination based on multiple social identities or positions [40,41]. Recent development on tools to identify intersectional discrimination, the Intersectional Anticipated Discrimination Scale [42] is a useful tool to understand the exposure to discrimination of hidden workers aged more than 45 years. We have integrated the questions into the survey together with the validated Workplace Age Discrimination Scale (WADS) [43]. These instruments were selected for their ability to capture multidimensional and intersectional experiences of discrimination and will be complemented by demographic variables (eg, gender, ethnicity, country of birth, education, employment history, income, and household structure) to allow analysis of intersectional effects.

Workplace Social Capital (WSC) is defined as a workplace resource that concerns employees’ perceptions regarding trust, reciprocity (cognitive WSC), and network interactions (structural WSC) that exist both among peers (bonding WSC) and among individuals across different hierarchical levels or organizations (bridging WSC) [44-46]. WSC is an important indicator to understand the relationship between relational aspects and an individual’s attitudes toward entering/re-entering the workforce. The validated WSC index will be used in this survey [47]. Together, these validated measures and contextual variables provide a robust methodological framework for multivariate and intersectional analyses. Furthermore, all instruments have demonstrated strong psychometric properties in aging worker populations, with internal consistency (Cronbach α) typically exceeding 0.80, ensuring reliability for this study cohort [32].

### Phase 2: Interview

The qualitative phase will be informed by the survey results from phase 1, in line with the explanatory sequential mixed methods design. Based on the quantitative findings from phase 1, a purposive subsample of approximately 30 participants will be invited to take part in in-depth qualitative interviews (September 2025-February 2026). The final number was determined by the need to ensure representation of key intersectional subgroups identified in the quantitative analysis (eg, combinations of minoritized status, condition or impairment, and citizenship status) and to reach thematic saturation.

From Facebook (Meta Platforms, Inc) advertisements, we will be recruiting participants to approximately reflect the characteristics of the aging populations facing multiple and intersecting barriers to entering or re-entering the workforce. The semistructured interview guide will be developed and piloted with 1-2 participants to refine questions. Semistructured interviews will be conducted via Zoom (Zoom Communications Inc). Zoom recordings are stored temporarily on the La Trobe University–approved encrypted cloud. Files are downloaded within 24 hours, deleted from the cloud, and retained on the university’s secure server. Access is restricted to the lead researcher and one authorized data manager under institutional data-handling agreements.

We will recruit interview participants from advertisements or links distributed through a range of platforms and networks, as well as through local lay coresearchers. We will rely on participant self-identification of citizenship status and condition or impairment. Posters, advertisements, and snowball sampling will target those who lack resources or technology to be recruited via online messages. They can contact us by telephone or email.

Respondents can choose to have their interviews by remote video methods, lasting approximately 60-90 minutes, by telephone, or in person (where feasible) to accommodate varying access needs. Interviews will be recorded and professionally transcribed. Attention will be given to making the interviews fully accessible and inclusive, and all researchers will be vigilant to the participant’s needs, such as requiring frequent breaks. All potential participants will be informed about the study in plain English (read to them if needed) and told that interviews will be in English by default. Plain-English language consent forms are provided to ensure that participants are able to give fully informed consent. The interview guide will be informed by the survey results (phase 1) and will cover the same key domains (Table 1), with flexibility to explore emergent themes.

**Table 1:** Variables collected from the survey on hidden workers.

Variables		Hidden workers	Current workers
**Actions**			
	Job search undertaken	✓	✓
	Engaged in learning	✓	✓
	Adapted climate impact mitigation strategies	✓	✓
**Intentions**			
	Willing to work	✓	
**Attitudes**			
	Degree of discouragement	✓	
	Passive or active job seekers	✓	
	Attitudes to learning	✓	✓
**Work history**			
	Work history	✓	✓
	Workplace discrimination	✓	✓
	Workplace social capital	✓	✓
**Health and well-being**			
	Long-term health conditions	✓	✓
	Subjective health status	✓	✓
	Mental health well-being	✓	✓
	Quality of life indicators	✓	✓
	Everyday discrimination index	✓	✓
**Conditions**			
	At-risk living conditions	✓	✓
	Childcare needs	✓	✓
	Other caregiving needs	✓	✓
	Household living arrangement	✓	✓
	Household income	✓	✓
**External contexts **			
	Impact of the COVID-19 pandemic	✓	✓
	Housing crisis	✓	✓
	Supportive workplace policies	✓	
**Assets **			
	Social capital (support networks)	✓	✓
	Access to learning	✓	✓
	Community assets	✓	✓

Prior to data collection, the interview guide will be pilot tested with 2-3 participants representative of the target population to ensure clarity, cultural appropriateness, and relevance. Feedback from the pilot will be used to refine question wording, sequence, and prompts. This approach will provide the qualitative phase that effectively expands upon and contextualizes the statistical patterns identified in the quantitative analysis.

The data will be retained for 5 years, and only the lead researcher will have access to the raw data. They will be asked about work and family biographies, their experience in the labor market, barriers to entering the job market, and health. Transcripts will be deidentified by removing names and potentially identifiable information before being analyzed thematically using NVivo qualitative software (Lumivero [33]), following Braun and Clarke’s [34] 6-phase framework for thematic analysis.

### Data Analysis: Quantitative Analysis

A descriptive statistical summary will be provided. More in-depth analysis will be conducted using Stata 18 (StataCorp LLC [48]), incorporating all variables in the dataset to address the research questions:

1. How do outcomes, including resource access, quality of life, physical and mental health, and social networks, and their trajectories differ between aging hidden workers and aging nonhidden workers across subgroups defined by minoritized status, condition or impairment, and citizenship status, as well as intersectional combinations of these characteristics?
2. To what extent are differences in outcomes and trajectories between aging hidden and nonhidden workers associated with healthy aging indicators?
3. What individual, structural, and contextual factors contribute most to between- and within-group disparities in outcomes and trajectories among aging workers?

Data analysis will be conducted in 3 stages, aligned with the research questions. For RQ1, descriptive statistics will compare key outcomes between aging hidden and nonhidden workers, followed by multiple regression models incorporating interaction terms (eg, group×minoritized status, condition or impairment, and citizenship) to assess intersectional effects. For RQ2, healthy aging indicators will be added to the models to examine their explanatory role in observed group differences. For RQ3, we will apply inequality decomposition techniques (eg, Blinder–Oaxaca) and variable importance ranking to quantify the contribution of individual, structural, and contextual factors to between- and within-group disparities.

Latent class models will be estimated via generalized structural equation modeling in Stata 18 using Akaike Information Criterion and Bayesian Information Criterion and entropy for class enumeration. Blinder–Oaxaca decompositions will quantify between-group disparities. Variable-importance ranking will use dominance analysis and Shapley Additive Explanations values derived from random forest models.

The final analytic sample comprised 1166 respondents. Missing data were assessed using Stata. Overall item nonresponse across core outcomes and exposures was low (typically <3%). Higher missingness observed in certain modules reflected structural missingness from survey branching (ie, respondents not eligible for those items). Analyses will be conducted within the appropriate universe for each item; structural missings will not be imputed. Where item nonresponse for a key variable exceeds ~5% among eligible respondents, we will perform multiple imputation under missing at random (with auxiliary variables) and compare against complete-case results, alongside prespecified missing not at random pattern-mixture sensitivity checks.

### Qualitative Analysis

Theoretical approach

The qualitative phase will apply the IHHIF as an analytic scaffold to explore how structural conditions, intersecting identities, and cognitive-behavioral processes jointly shape the lived experiences of ageing hidden workers.

#### Analytic Approach

Interviews will be transcribed verbatim and analyzed thematically following the 6-phase process—familiarization, coding, theme development, review, definition, and reporting [34] —using NVivo 12 software. Coding and interpretation will be guided by the IHHIF domains, while remaining open to emergent, inductive insights.

1. Structural determinants (Commission on Social Determinants of Health layer): Codes will first identify macro- and mesolevel conditions influencing participants’ employment trajectories, such as labor-market policies, welfare and pension rules, employer practices, digital access, and regional or industry-specific constraints. Attention will be given to how these structural factors interact with participants’ material circumstances (housing, income, transport, and health service access) to enable or constrain participation.

2. Intersecting identities: Next, analytic focus will shift to how social positions—age, gender, class, ethnicity, cultural background, health, and caregiving—combine to produce unique configurations of advantage or exclusion. The analysis will examine how participants describe the simultaneous effects of these identities (eg, being an aging migrant woman caring for grandchildren) and how these combinations influence perceptions of employability, discrimination, and well-being.

3. Cognitive–behavioral mediators (Social Cognitive Career Theory layer): Themes will then explore individual-level mechanisms, such as self-efficacy, outcome expectations, attitudes toward aging and work, intentions to re-enter employment, and enacted behaviors (job search, learning, and adaptation). Narrative coding will examine how experiences of rejection, support, or discrimination shape these internal processes over time.

4. Health and employment outcomes: Analyses will integrate descriptions of participants’ health trajectories (physical, mental, and emotional) and employment status to map how cumulative disadvantage manifests in outcomes. Links will be traced between structural barriers, identity intersections, and cognitive mediators to illustrate the pathways through which inequities develop.

5. Assets and feedback loops: Finally, data will be reviewed for evidence of resilience and enabling resources—such as community belonging, informal work, volunteering, retraining, or supportive employers—and for feedback mechanisms where improved health or social capital facilitates re-engagement.

#### Interpretive Synthesis

Using a matrix-coding approach, themes will be compared across subgroups (eg, gender, Culturally and Linguistically Diverse status, and caregiving role) to identify recurring intersectional patterns. A conceptual map will then be produced linking thematic domains to the IHHIF components (Structural Determinants→Identities→Cognitive Mediators→Outcomes→Assets), providing an integrated qualitative representation of how multilevel forces shape hidden workers’ health and employment experiences.

To enhance rigor, 2 researchers will independently code a subset of transcripts, and discrepancies will be resolved through reflexive discussion. Memos will document analytic decisions and reflexivity regarding researchers’ positionalities. The final synthesis will inform integration with quantitative findings in phase 3, ensuring the qualitative insights directly illuminate the mechanisms hypothesized by the IHHIF.

### Phase 3: Integration of Quantitative and Qualitative Findings

**Figure 2:**
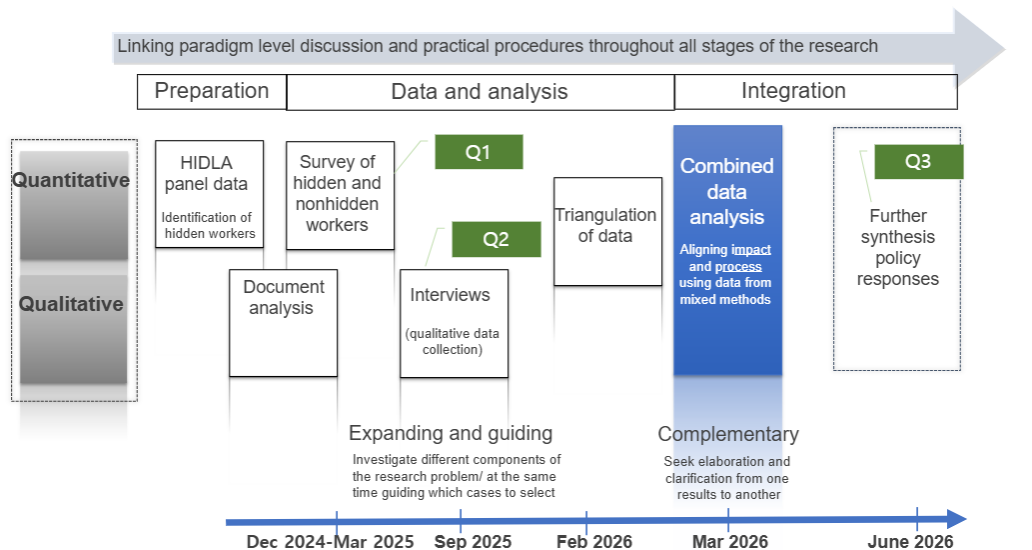
Mixed methods research schematic.

As shown in Figure 2, this phase will integrate outputs from phase 1 (survey) and phase 2 (interviews) to provide a comprehensive synthesis (from February to June 2026).

Triangulation is defined as the act of seeking convergence of results of the same research question from different methods to increase the validity of results. A main advantage of triangulation is its attempts to validate, or enhance the accuracy of, research findings by comparing different sorts of data on the same topic, with qualitative findings substantiating quantitative findings. Moreover, if convergence is not achieved, quantitative and qualitative data can be put into dialogue with each other through the use of participant review groups, who can offer improved interpretations on both sets of findings [47].

Complementarity, in contrast, allows scholars to elaborate and enhance results from one method with results from another method, with attention to multiple facets of the same question. For instance, combining qualitative data with quantitative results contextualizes hidden female workers’ experiences by highlighting how labor market practices create a caustic environment to be kept hidden. A complementary intersectional mixed methods research approach is useful for research on multiply marginalized hidden workers, specifically because it uses an intersectional framework to both identify patterns relevant to hidden workers and explain why these patterns may exist in a single study.

Implementing these strategies within a single study could provide a comprehensive understanding of the desired needs and services for this client group while also providing clear direction to the center for addressing gaps. To date, the scholarship on intersectionality theory and the literature on mixed methods research have largely developed in silos, with limited interdisciplinary conversation about the benefits of their integration. Intersectionality scholars have elucidated some advantages of combining statistical approaches with in-depth narratives [32-34]; however, in practice, few studies have fully realized what is possible in intersectional mixed methods research, especially regarding tackling complex questions concerning multiply marginalized hidden workers.

Integration will be conducted collaboratively by both qualitative and quantitative researchers to ensure balanced interpretation. The final outcome of this phase will be a synthesis report, policy recommendations, and community briefs co-developed with the Project Advisory Group and stakeholders. Thus, this study aimed to highlight how intersectional mixed methods research could be used for several purposes to advance research on hidden workers.

### Disseminations

Communications will be achieved through the participants’ preferred method of communication. We will tailor to our key audiences, which emphasize practical solutions and implementation. The dissemination plan will be determined with our Project Advisory Group and through our cocreation meetings, with an indicative timeline to ensure early, interim, and final updates are shared in sequence. Findings will be disseminated through peer-reviewed publications, national, and international conferences.

Overall synthesis will provide an executive overview for easy assimilation by policymakers and practitioners. This will indicate where changes to health and social care policy and practice are likely to be most effective, with targeted briefings and roundtable discussions to maximize uptake. An overview of findings and ideas for outputs will be presented to participating communities more widely via collaborator platforms to give them the opportunity to reflect upon and interrogate researchers’ interpretations and analysis of the data, and their feedback will be incorporated where appropriate.

Findings and ideas for outputs will also be distributed through trusted community channels, such as places of worship, trusted religious leaders, and community champions. This will enable broader community input into the final project outputs. All findings will be publicly available via our website in accessible forms for lay consumption. Confidentiality will be maintained throughout, with any potentially identifying information removed before dissemination.

### Ethical Considerations

#### Ethical Approval

Ethics approval was granted by the La Trobe University Human Research Ethics Committee (HEC 23459). All participants provide informed consent prior to participation. Survey data are anonymous, interview data are deidentified, and all files are stored on encrypted, access-controlled university servers. All procedures complied with the ethical standards of the institutional and national research committees and with the principles outlined in the Declaration of Helsinki.

Informed Consent All participants were provided with a detailed plain language statement outlining the study purpose, procedures, voluntary participation, and their right to withdraw at any time without penalty. Informed consent was obtained from all participants prior to data collection. For online surveys, consent was documented through an electronic consent form embedded at the start of the questionnaire (participants could not proceed without agreeing).

Privacy and Confidentiality Data were collected and stored in accordance with institutional policies and applicable privacy regulations. Identifiable information was not collected unless explicitly required for follow-up procedures and was stored separately from survey/interview responses. All data were deidentified prior to analysis. Only authorized members of the research team had access to the encrypted, password-protected data repository. No identifiable information will appear in publications or presentations.

Compensation Participants did/did not receive compensation for their involvement in the survey, but interview participants were compensated with an AUD $25 (US $16.55) Coles voucher.

## Results

Funding for this study was awarded by La Trobe University in June 2024 (HEC23459). The online survey (phase 1) was launched in December 2024 and completed in March 2025, achieving a total sample of 1166 participants aged 45 years and older across all Australian states and territories. Recruitment for the qualitative interview phase (phase 2) commenced in September 2025 and is ongoing. Data cleaning for the survey responses and preliminary coding has been completed. Integration of survey and interview findings (phase 3) will occur between September and February 2026. Papers reporting the quantitative and qualitative findings are expected to be submitted to peer-reviewed journals in 2026.

## Discussion

### Anticipated Findings and Potential Impact

In undertaking this study, we will fill a gap in the evidence about the hidden workers in aging Australia. We expect to contribute considerable new knowledge through our mixed methods approach, aligned with national priorities on workforce participation and skills, including the current Australian workforce and aging strategies [49]. Drawing on the survey data, we will quantify issues, such as those relating to access to labor market resources, social networks of support, and discrimination and marginalization. However, we are particularly interested in the strengths and assets that have improved our participants’ capacity to cope with their unemployment or underemployment to identify scalable supports and pathways that can be piloted and, if effective, implemented nationally with employer and community partners. We will track uptake through policy citations, stakeholder workshops, and media mentions within 12 months post publication as indicators of impact.

We believe this is important, as many health and well-being challenges, such as those faced by minority ethnic groups at the intersection with chronic conditions or impairments and insecure citizenship status, can be mitigated by adjustments to health and social care services, policy, and delivery; formal networks such as community health services; and informal networks such as family and friends. We expect to provide recommendations for these adjustments and for potential interventions through this mixed methods analysis, translated into policy briefs, practitioner guidance, and community toolkits that can support rapid adoption by government, providers, and employers. We may also design some simple interventions ourselves, including low-cost, co-designed pilots targeting identified barriers, such as digital skills gaps, flexible work design, or navigation support. To attempt to tease out the impact of the aging hidden workers, we will (1) model relationships between mediating variables (including social network features) and health and social outcomes (and consider indicative economic and workforce implications of reduced early exit and improved re-entry) and (2) explore participants’ current and recent experiences and labor market access.

It is anticipated that the research will provide new information on the interventions, support, and individual factors that assist the aging hidden workers, with relevance to other aging economies facing similar hidden workforce challenges. The academic research will not only help inform how the Australian government can improve its interventions but also will provide new information on how to prevent people from having to leave work and extend working lives. This research will inform current and future welfare-to-work and job retention initiatives and improve their effectiveness in helping aging people extend their healthy working lives.

### Building on Prior Research

This study builds directly on emerging work examining the visibility and experiences of hidden workers [8,36,50]. Foundational Australian research has shown the long-term impacts of unemployment and care-related labor interruptions on financial security, labor force attachment, and health [19,20]. International scholarship has also emphasized the need to address structural conditions shaping the opportunities of older, disadvantaged workers [37]. By integrating intersectional mixed methods data, this study will extend existing research by providing deeper, multidimensional evidence on how overlapping disadvantages shape employment trajectories among those aged 45 years and older.

### Strengths and Limitations

We will provide rich quantitative and qualitative data, with an adequate sample size for qualitative interviews, providing in-depth information through quota sampling. Our research approach will be key to cocreating our outputs with relevant stakeholders. This will include members of the populations we hope will benefit, hidden workers, industry, communities, and the government. This will ensure outputs that have real credibility, real-world relevance, and value can be implemented and are sustainable.

Nonetheless, the study has its limitations. First, the survey, being primarily digital, will exclude people with poor access to the digital world, although we do offer alternatives, such as phone-based interviews. Second, providing participant information forms in the English language only may result in the exclusion of individuals with limited English proficiency. Potential self-selection and recall bias may occur, and social desirability effects could lead to underreporting of discrimination. Triangulation with qualitative data will help mitigate these.

Finally, although the ideal study design would have a longitudinal survey, we cannot undertake repeated surveys due to various limitations. However, rigorous synthesis of the multiple types of data we produce and a reflexive approach to biases should help to contextualize findings within these limitations.

### Conclusions

Current understandings of hidden workers in the aging workforce are limited with regard to the intersections of various minoritized population subgroups. Existing inequities may have worsened, and public and policy awareness of this exacerbation provides an opportunity for change. This study, using an intersectional assets-based approach and drawing on participatory and mixed methods, aims to fill a gap in the evidence to help inform changes that reduce the health inequities of hidden workers in an aging context.

## Appendix

Multimedia Appendix 1 The Hidden Workers in Ageing Australia Survey.

## Abbreviations

ABS: Australian Bureau of Statistics
DEWR: Department of Employment and Workplace Relations
IHHIF: Intersectionality‑based Hidden Workers Health Inequality Framework
ILO: International Labour Organization
OECD: Organisation for Economic Co-operation and Development
WADS: Workplace Age Discrimination Scale
WSC: Workplace Social Capital

## Data Availability

The datasets generated or analyzed during this study are not publicly available due to ethics requirements but are available from the corresponding author under ethics-approved conditions.
