# Supplementary material for: Hidden Workers in Aging Australia: Protocol of Intersectionality-Informed Mixed Methods Study
Source: JMIR Res Protoc. 2025 Dec 31;14:e83401. doi: 10.2196/83401 (PMC12805321; doi:10.2196/83401)
Supplement: Multimedia Appendix 1 [file resprot_v14i1e83401_app1.docx]

Hidden Workers in Aging Australia

(Ethics approval no. HEC23459)

Can you tell me what age you are?

|  |
| --- |

Which of this best describes your current situation?

1. Not in paid employment, not looking for paid work
2. Employed, 0-30 hours per week
3. Employed, 30-43; hours a week
4. Unemployed
5. Permanently unable to work
6. Retired
7. Prefer not to say
8. None of the above

Are you willing to work?

1. Yes
2. No

What are your reasons for being unable to work?

1. Serious illness that requires treatment
2. Physical injury requiring long recovery periods
3. Severe mental health issues.
4. Other

What is your main reason for working part time hours rather than full time?

1. Own illness or disability
2. Caring for family members
3. Other personal or family responsibility
4. Going to school, college, university
5. Could not find full time work
6. Prefer part time work
7. Involved in voluntary work
8. Attracted to pay premium
9. Welfare payments or pension may be reduced
10. None of these
11. Other

Which of these describes your health reasons for part-time?

1. Work is physically demanding
2. Mentally stressful at work
3. Cognitive capacity, memory issues
4. Loss of hearing, vision
5. Mobility
6. Reduced strength, frailty
7. General fatigue
8. Chronic pain
9. None of these
10. other

Please rate below sentences that starts with, "My current job ~

|  | 1 | 2 | 3 | 4 | 5 |
| --- | --- | --- | --- | --- | --- |
| is secure. | ❏ | ❏ | ❏ | ❏ | ❏ |
| is appropriately paid. | ❏ | ❏ | ❏ | ❏ | ❏ |
| treats me fair and equal. | ❏ | ❏ | ❏ | ❏ | ❏ |
| provides me with adequate  benefits | ❏ | ❏ | ❏ | ❏ | ❏ |
| is flexible so I can accommodate my needs | ❏ | ❏ | ❏ | ❏ | ❏ |

Which of the following best describes your contract of employment for that job? Self-employed? Employed on a fixed-term contract? Employed on a casual basis? Employed on a permanent or ongoing basis? Or employed on some other type of contract?

1. Self-employed
2. Employed on a fixed-term contract
3. Employed on a casual basis
4. Employed on a permanent or ongoing basis
5. Other
6. Don’t know

Are you willing to work more hours than you are currently working?

1. Yes
2. No

Which best describes your retirement status?

1. Permanently retired
2. Transitioning into retirement by reducing my hours
3. I move in and out of retirement
4. Prefer not to say

Why did you retire? (You may choose more than one)

1. Became eligible for age pension
2. Superannuation rules made it financially advantageous to retire at that time
3. Ill health of other family member
4. Own ill health
5. Ill health of spouse/partner
6. To have more personal/leisure time
7. Made redundant/dismissed/had no choice
8. Could not find another job
9. Pressure from employer or others at work
10. Partner had just retired or was about to retire
11. Reached compulsory retirement age
12. Spouses/ partners income enabled me to retire
13. Spouse/partner wanted me to retire
14. To spend more time with other family
15. To spend more time with spouse/partner
16. Offered reasonable financial terms to retire
17. Fed up with working/work stresses, demands
18. None of these
19. other

Which of these describes your health reasons for retirement?

1. Work is physically demanding
2. Mentally stressful at work
3. Cognitive capacity, memory issues
4. Loss of hearing, vision
5. Mobility
6. Reduced strength, frailty
7. General fatigue
8. Chronic pain
9. None of these
10. Other

Have you thought about re-entering the paid workforce since retiring?

1. Yes
2. No, but I would consider it
3. No, I wouldn’t consider it
4. Prefer not to say

If you are offered one of below, would you take it?

|  | Yes | No |
| --- | --- | --- |
| A secure job | ❏ | ❏ |
| An appropriately paid job | ❏ | ❏ |
| A job with fair and equal treatment | ❏ | ❏ |
| A job with adequate benefits | ❏ | ❏ |
| A flexible job that accommodates your needs | ❏ | ❏ |

Why do you want to re-enter the workforce?

1. Was bored/needed something to do
2. I no longer needed to care for the person I retired to care for
3. Do not like being retired
4. Death of spouse/partner
5. Employer/business needed me
6. Exciting new possibilities came up that I could not resist
7. Pressure from other family member
8. Needed money
9. Own health improved
10. Separation from partner/divorce
11. Other

Do you currently receive any income from the government in the form of a benefit, pension or allowance?

1. Age pension
2. Disability support pension
3. Jobseeker payment
4. Parenting payment
5. Other
6. None

What are (or will be) the sources of income in retirement for your household? (Please check all that apply)

1. Age Pension
2. Part-time work
3. Super fund account-based pension / allocated pension
4. Defined benefit pension
5. Lifetime annuity
6. Term deposits
7. Dividends from shares
8. Rent from investment property
9. Selling down investments
10. Income from family / private business
11. Don't know
12. Other

Please let me know your employment status for the PREVIOUS 4 years

|  | Not in paid employment, not looking for paid work | Employed, 0-30 hr/week | Employed, 30+ hr/week | Unemployed, Looking for paid work | Unable to work | Retired |
| --- | --- | --- | --- | --- | --- | --- |
| July 2022 | ❏ | ❏ | ❏ | ❏ | ❏ | ❏ |
| July 2021 | ❏ | ❏ | ❏ | ❏ | ❏ | ❏ |
| July 2020 | ❏ | ❏ | ❏ | ❏ | ❏ | ❏ |
| July 2019 | ❏ | ❏ | ❏ | ❏ | ❏ | ❏ |

Which of the following best describes your contract of employment for that job? Self-employed? Employed on a fixed-term contract? Employed on a casual basis? Employed on a permanent or ongoing basis? Or employed on some other type of contract?

|  | Self-employed | Employed on a fixed-term contract | Employed on a casual basis | Employed on a permanent or ongoing basis | Digital platform worker (Uber) | Other |
| --- | --- | --- | --- | --- | --- | --- |
| 2019 | ❏ | ❏ | ❏ | ❏ | ❏ | ❏ |
| 2020 | ❏ | ❏ | ❏ | ❏ | ❏ | ❏ |
| 2021 | ❏ | ❏ | ❏ | ❏ | ❏ | ❏ |
| 2022 | ❏ | ❏ | ❏ | ❏ | ❏ | ❏ |

What is your current occupation? If currently unemployed, please choose your previous occupation.

1. Managers
2. Professionals
3. Technicians and trades workers
4. Community and personal service workers
5. Clerical/administrative workers
6. Sales workers
7. Machinery operations and drivers
8. Labourers
9. Student
10. Other
11. Never employed
12. Self-employed

What is the industry of your current employment (if currently employed). If currently unemployed, please choose your previous industry.

1. Construction, manufacturing, mining
2. Agriculture, forestry and fishing, transport and storage, electricity/gas/water supply
3. Government, education, communication, finance, and insurance services
4. Wholesale/retail trade, hospitality/tourism/accommodation, perperty and business services
5. Cultural recreational/persona/health and community services
6. Other
7. Never worked

Have you ever participated in the job-seeking activity in the last four weeks?

1. Yes
2. No

If yes, have you undertaken any of the following activities? CHOOSE ALL THAT APPLY

1. Contacting an employer directly or having a job interview
2. Contacting a public or private employment agency
3. Asking friends or relatives for opportunities
4. Visit a school or university employment center
5. Submitting resumes or filling out applications
6. Placing or answering job advertisements
7. Checking union or professional registers
8. Some other means of active job search

Please indicate using the slider to show how strongly you agree with the below sentences.

|  |  |
| --- | --- |
| I am discouraged to look for jobs | ❏ |

Please indicate how strongly you agree or disagree with the statement “I am confident I will be able to get a fulltime job within the next six months”

1. Strongly agree
2. Agree
3. Neither agree nor disagree
4. Disagree
5. Strongly disagree

To understand the contextual challenges that can limit employment, please mark your TOP THREE reasons. You will be able to rank your choices after you choose them.

1. There is lack of jobs in the area I live.
2. There is lack of jobs in the industry I’d like to work in.
3. I’m not the right ‘fit’ for many organizations.
4. I lack qualifications.
5. I lack experience.
6. Pension rules dictates that I can’t work/work more hours.
7. I can’t find work that I can balance my disability/ health challenges.
8. I can’t find work that I can balance with my caregiving responsibilities.
9. I’m scared of failure.

- There is lack of jobs in the area I live. __________
- There is lack of jobs in the industry I’d like to work in. __________
- I’m not the right ‘fit’ for many organizations. __________
- I lack the qualifications employer’s demand. __________
- I lack the experience that employer demands. __________
- Pension rules dictates that I can’t work/work more hours. __________
- I can’t find work that I can balance my disability/ health challenges. __________
- I can’t find work that I can balance with my caregiving responsibilities. __________
- I’m scared of failure. __________

What triggered a change in your circumstance leading you to look for work? Please mark TOP THREE reasons. You will be able to rank your choices after you choose them.

1. Change in family members
2. Change in healthcare costs
3. Change in my outlook/expectations
4. Mental health improved
5. Immigration status change
6. New skills/experience/qualifications
7. Supportive employer practices
8. Supportive government policies
9. Change in economic outlook

- Change in family members __________
- Change in healthcare costs __________
- Change in my outlook/expectations __________
- Mental health improved __________
- Immigration status change __________
- New skills/experience/qualifications __________
- Supportive employer practices __________
- Supportive government policies __________
- Change in economic outlook __________

Have you taken any of the following actions to find paid employment? Please mark maximum THREE reasons. You will be able to rank your choices after you choose them.

1. Developed my digital skills
2. Assessed my transferable skills (e.g., skills that can be taken from one industry to another)
3. Developed my soft skills
4. Obtained (more) technical qualifications
5. Undertook work experience (paid or unpaid)
6. Built my professional network
7. Changed personal image/appearance
8. Learned about emerging technologies (AI, virtual/extended reality, cloud, blockchain, etc.)
9. Changed home location (e.g., moved to a new city)
10. Re-enrolled into education to attain (more) academic qualifications
11. Learned a new language
12. None of the above
13. Not taken any actions

- Developed my digital skills __________
- Assessed my transferable skills (e.g., skills that can be taken from one industry to another) __________
- Developed my soft skills __________
- Obtained (more) technical qualifications __________
- Undertook work experience (paid or unpaid) __________
- Built my professional network __________
- Changed personal image/appearance __________
- Learned about emerging technologies (AI, virtual/extended reality, cloud, blockchain, etc.) __________
- Changed home location (e.g., moved to a new city) __________
- Re-enrolled into education to attain (more) academic qualifications __________
- Learned a new language __________
- None of the above __________
- Not taken any actions __________

What are the conditions of employment that you find supportive of your situation? Please mark your TOP THREE reasons.

1. Flexible working
2. Training/re-skilling for current and future jobs
3. Supportive leadership team
4. Policies supporting work-life balance
5. Adoption of digital technologies
6. 5-10 days additional paid leave
7. Supportive physical workplace environment
8. Peer support network
9. Free transportation to commute to work
10. Culture champions for inclusion and diversity
11. Policies supporting diversity
12. Out-of-the-box compensation benefits
13. Re-designing work
14. Education/awareness programs to champion cultural change
15. Subsidized childcare/on-site crèche
16. Unpaid long-term leave

- Flexible working __________
- Training/re-skilling for current and future jobs __________
- Supportive leadership team __________
- Policies supporting work-life balance __________
- Adoption of digital technologies __________
- 5-10 days additional paid leave __________
- Supportive physical workplace environment __________
- Peer support network __________
- Free transportation to commute to work __________
- Culture champions for inclusion and diversity __________
- Policies supporting diversity __________
- Out-of-the-box compensation benefits __________
- Re-designing work __________
- Education/awareness programs to champion cultural change __________
- Subsidized childcare/on-site crèche __________
- Unpaid long-term leave __________

Please complete sentences with the choices that best describe your experience in current social challenges.

|  | More difficult | About the same | Less difficult |
| --- | --- | --- | --- |
| Since the Covid-19 pandemic began, finding work/working more hours have become : | ❏ | ❏ | ❏ |
| Housing crisis makes finding work/working more hours: | ❏ | ❏ | ❏ |
| Inadequate public transport makes finding work/working more hours | ❏ | ❏ | ❏ |
| The ageist stereotype for older person working makes finding work/working more hours: | ❏ | ❏ | ❏ |

Have you ever found a job through your networks?

1. Yes
2. No

These questions are about experiences related to who you are. This includes both how you describe yourself and how others might describe you. For example, your skin color, ancestry, nationality, religion, gender, sexuality, age, weight, disability or mental health issue, and income. Choose the answer that best describes your answer to the sentence below.

|  | Strongly disagree | Disagree | Neither agree or disagree | Agree | Strongly agree |
| --- | --- | --- | --- | --- | --- |
| Because of who I am, a doctor or nurse, or other health care provider might treat me poorly. | ❏ | ❏ | ❏ | ❏ | ❏ |

What was (were) the reason(s) for this experience? Multiple responses are possible.

1. Your ancestry or nationality
2. Your gender
3. Your age
4. Your religion
5. Your height
6. Your weight
7. Some other aspect of your physical appearance
8. Disability or mental health issues
9. Your sexual orientation
10. Your education or income level
11. Other __________

Choose the answer that best describes your answer to the sentence below.

|  | Strongly disagree | Disagree | Neither agree or disagree | Agree | Strongly agree |
| --- | --- | --- | --- | --- | --- |
| Because of who I am, I might have trouble finding or keeping a job. | ❏ | ❏ | ❏ | ❏ | ❏ |

What was (were) the reason(s) for this experience? Multiple responses are possible.

1. Your ancestry or nationality
2. Your gender
3. Your age
4. Your religion
5. Your height
6. Your weight
7. Some other aspect of your physical appearance
8. Disability or mental health issues
9. Your sexual orientation
10. Your education or income level
11. Other __________

Choose the answer that best describes your answer to the sentence below.

|  | Strongly disagree | Disagree | Neither agree or disagree | Agree | Strongly agree |
| --- | --- | --- | --- | --- | --- |
| Because of who I am, I might have trouble getting an apartment or house. | ❏ | ❏ | ❏ | ❏ | ❏ |

What was (were) the reason(s) for this experience? Multiple responses are possible.

1. Your ancestry or nationality
2. Your gender
3. Your age
4. Your religion
5. Your height
6. Your weight
7. Some other aspect of your physical appearance
8. Disability or mental health issues
9. Your sexual orientation
10. Your education or income level
11. Other __________

Choose the answer that best describes your answer to the sentence below.

|  | Strongly disagree | Disagree | Neither agree or disagree | Agree | Strongly agree |
| --- | --- | --- | --- | --- | --- |
| I worry about being treated unfairly by a teacher, supervisor, or employer. | ❏ | ❏ | ❏ | ❏ | ❏ |

What was (were) the reason(s) for this experience? Multiple responses are possible.

1. Your ancestry or nationality
2. Your gender
3. Your age
4. Your religion
5. Your height
6. Your weight
7. Some other aspect of your physical appearance
8. Disability or mental health issues
9. Your sexual orientation
10. Your education or income level
11. Other __________

Choose the answer that best describes your answer to the sentence below.

|  | Strongly disagree | Disagree | Neither agree or disagree | Agree | Strongly agree |
| --- | --- | --- | --- | --- | --- |
| I may be denied a bank account, loan, or mortgage because of who I am. | ❏ | ❏ | ❏ | ❏ | ❏ |

What was (were) the reason(s) for this experience? Multiple responses are possible.

1. Your ancestry or nationality
2. Your gender
3. Your age
4. Your religion
5. Your height
6. Your weight
7. Some other aspect of your physical appearance
8. Disability or mental health issues
9. Your sexual orientation
10. Your education or income level
11. Other __________

Choose the answer that best describes your answer to the sentence below.

|  | Strongly disagree | Disagree | Neither agree or disagree | Agree | Strongly agree |
| --- | --- | --- | --- | --- | --- |
| I worry about being harassed or stopped by police or security. | ❏ | ❏ | ❏ | ❏ | ❏ |

What was (were) the reason(s) for this experience? Multiple responses are possible.

1. Your ancestry or nationality
2. Your gender
3. Your age
4. Your religion
5. Your height
6. Your weight
7. Some other aspect of your physical appearance
8. Disability or mental health issues
9. Your sexual orientation
10. Your education or income level
11. Other __________

Choose the answer that best describes your answer to the sentence below.

|  | Strongly disagree | Disagree | Neither agree or disagree | Agree | Strongly agree |
| --- | --- | --- | --- | --- | --- |
| Because of who I am, people might try to attack me physically. | ❏ | ❏ | ❏ | ❏ | ❏ |

What was (were) the reason(s) for this experience? Multiple responses are possible.

1. Your ancestry or nationality
2. Your gender
3. Your age
4. Your religion
5. Your height
6. Your weight
7. Some other aspect of your physical appearance
8. Disability or mental health issues
9. Your sexual orientation
10. Your education or income level
11. Other __________

Choose the answer that best describes your answer to the sentence below.

|  | Strongly disagree | Disagree | Neither agree or disagree | Agree | Strongly agree |
| --- | --- | --- | --- | --- | --- |
| I expect to be pointed at, called names, or harassed when in public. | ❏ | ❏ | ❏ | ❏ | ❏ |

What was (were) the reason(s) for this experience? Multiple responses are possible.

1. Your ancestry or nationality
2. Your gender
3. Your age
4. Your religion
5. Your height
6. Your weight
7. Some other aspect of your physical appearance
8. Disability or mental health issues
9. Your sexual orientation
10. Your education or income level
11. Other __________

Choose the answer that best describes your answer to the sentence below.

|  | Strongly disagree | Disagree | Neither agree or disagree | Agree | Strongly agree |
| --- | --- | --- | --- | --- | --- |
| I fear that I will have a hard time finding friendship or romance because of who I am. | ❏ | ❏ | ❏ | ❏ | ❏ |

What was (were) the reason(s) for this experience? Multiple responses are possible.

1. Your ancestry or nationality
2. Your gender
3. Your age
4. Your religion
5. Your height
6. Your weight
7. Some other aspect of your physical appearance
8. Disability or mental health issues
9. Your sexual orientation
10. Your education or income level
11. Other __________

These questions are about experiences related to who you are. This includes both how you describe yourself and how others might describe you. For example, your skin color, ancestry, nationality, religion, gender, sexuality, age, weight, disability or mental health issue, and income. Because of who you are, have you...

|  | Never | Yes, but not in the past year | Yes, once or twice in the past year | Yes, many times in the past year |
| --- | --- | --- | --- | --- |
| Heard, saw, or read others joking or laughing about you (or people like you) | ❏ | ❏ | ❏ | ❏ |

What was (were) the reason(s) for this experience? Multiple responses are possible.

1. Your ancestry or nationality
2. Your gender
3. Your age
4. Your religion
5. Your height
6. Your weight
7. Some other aspect of your physical appearance
8. Disability or mental health issues
9. Your sexual orientation
10. Your education or income level
11. Other __________

Because of who you are, have you...

|  | Never | Yes, but not in the past year | Yes, once or twice in the past year | Yes, many times in the past year |
| --- | --- | --- | --- | --- |
| Been treated as if you are unfriendly, unhelpful, or rude | ❏ | ❏ | ❏ | ❏ |

What was (were) the reason(s) for this experience? Multiple responses are possible.

1. Your ancestry or nationality
2. Your gender
3. Your age
4. Your religion
5. Your height
6. Your weight
7. Some other aspect of your physical appearance
8. Disability or mental health issues
9. Your sexual orientation
10. Your education or income level
11. Other __________

Because of who you are, have you...

|  | Never | Yes, but not in the past year | Yes, once or twice in the past year | Yes, many times in the past year |
| --- | --- | --- | --- | --- |
| Been called names or heard/saw your identity used as an insult | ❏ | ❏ | ❏ | ❏ |

What was (were) the reason(s) for this experience? Multiple responses are possible.

1. Your ancestry or nationality
2. Your gender
3. Your age
4. Your religion
5. Your height
6. Your weight
7. Some other aspect of your physical appearance
8. Disability or mental health issues
9. Your sexual orientation
10. Your education or income level
11. Other __________

Because of who you are, have you...

|  | Never | Yes, but not in the past year | Yes, once or twice in the past year | Yes, many times in the past year |
| --- | --- | --- | --- | --- |
| Been treated as if others are afraid of you | ❏ | ❏ | ❏ | ❏ |

What was (were) the reason(s) for this experience? Multiple responses are possible.

1. Your ancestry or nationality
2. Your gender
3. Your age
4. Your religion
5. Your height
6. Your weight
7. Some other aspect of your physical appearance
8. Disability or mental health issues
9. Your sexual orientation
10. Your education or income level
11. Other __________

Because of who you are, have you...

|  | Never | Yes, but not in the past year | Yes, once or twice in the past year | Yes, many times in the past year |
| --- | --- | --- | --- | --- |
| Been stared or pointed at in public | ❏ | ❏ | ❏ | ❏ |

What was (were) the reason(s) for this experience? Multiple responses are possible.

1. Your ancestry or nationality
2. Your gender
3. Your age
4. Your religion
5. Your height
6. Your weight
7. Some other aspect of your physical appearance
8. Disability or mental health issues
9. Your sexual orientation
10. Your education or income level
11. Other __________

Because of who you are, have you...

|  | Never | Yes, but not in the past year | Yes, once or twice in the past year | Yes, many times in the past year |
| --- | --- | --- | --- | --- |
| Been told that you should think, act, or look more like others | ❏ | ❏ | ❏ | ❏ |

What was (were) the reason(s) for this experience? Multiple responses are possible.

1. Your ancestry or nationality
2. Your gender
3. Your age
4. Your religion
5. Your height
6. Your weight
7. Some other aspect of your physical appearance
8. Disability or mental health issues
9. Your sexual orientation
10. Your education or income level
11. Other __________

Because of who you are, have you...

|  | Never | Yes, but not in the past year | Yes, once or twice in the past year | Yes, many times in the past year |
| --- | --- | --- | --- | --- |
| Heard that you or people like you don't belong | ❏ | ❏ | ❏ | ❏ |

What was (were) the reason(s) for this experience? Multiple responses are possible.

1. Your ancestry or nationality
2. Your gender
3. Your age
4. Your religion
5. Your height
6. Your weight
7. Some other aspect of your physical appearance
8. Disability or mental health issues
9. Your sexual orientation
10. Your education or income level
11. Other __________

Because of who you are, have you...

|  | Never | Yes, but not in the past year | Yes, once or twice in the past year | Yes, many times in the past year |
| --- | --- | --- | --- | --- |
| Asked inappropriate, offensive, or overly personal questions | ❏ | ❏ | ❏ | ❏ |

What was (were) the reason(s) for this experience? Multiple responses are possible.

1. Your ancestry or nationality
2. Your gender
3. Your age
4. Your religion
5. Your height
6. Your weight
7. Some other aspect of your physical appearance
8. Disability or mental health issues
9. Your sexual orientation
10. Your education or income level
11. Other __________

Because of who you are, have you...

|  | Never | Yes, but not in the past year | Yes, once or twice in the past year | Yes, many times in the past year |
| --- | --- | --- | --- | --- |
| Been treated as if you are less smart or capable than others | ❏ | ❏ | ❏ | ❏ |

What was (were) the reason(s) for this experience? Multiple responses are possible.

1. Your ancestry or nationality
2. Your gender
3. Your age
4. Your religion
5. Your height
6. Your weight
7. Some other aspect of your physical appearance
8. Disability or mental health issues
9. Your sexual orientation
10. Your education or income level
11. Other __________

In the following questions, we are interested in the way other people have treated you AT YOUR WORKPLACE because of your age. Reflecting on your current and previous experience, how often do the following things happen to you?

|  | 1 | 2 | 3 | 4 | 5 |
| --- | --- | --- | --- | --- | --- |
| I have been passed over for a work role/task due to my age. | ❏ | ❏ | ❏ | ❏ | ❏ |
| My contributions are not valued as much due to my age. | ❏ | ❏ | ❏ | ❏ | ❏ |
| I have been given fewer opportunities to express my ideas due to my age. | ❏ | ❏ | ❏ | ❏ | ❏ |
| I have been evaluated less favourably due to my age. | ❏ | ❏ | ❏ | ❏ | ❏ |
| I receive less social support due to my age. | ❏ | ❏ | ❏ | ❏ | ❏ |
| I have been treated as though I am less capable due to my age. | ❏ | ❏ | ❏ | ❏ | ❏ |
| I have been treated with less respect due to my age. | ❏ | ❏ | ❏ | ❏ | ❏ |
| Someone has delayed or ignored my requests due to my age. | ❏ | ❏ | ❏ | ❏ | ❏ |
| Someone has blamed me for failures or problems due to my age. | ❏ | ❏ | ❏ | ❏ | ❏ |

Please rate below statements regarding workplace social capital, based on your current or previous (if currently unemployed) experience.

|  | Strongly disagree | Disagree | Neither agree nor disagree | Agree | Strongly agree | Not applicable |
| --- | --- | --- | --- | --- | --- | --- |
| Our supervisor treats us with kindness and consideration | ❏ | ❏ | ❏ | ❏ | ❏ | ❏ |
| Our supervisor shows concern for our rights as an employee | ❏ | ❏ | ❏ | ❏ | ❏ | ❏ |
| We have a 'we are together' attitude | ❏ | ❏ | ❏ | ❏ | ❏ | ❏ |
| People keep each other informed about work-related issues in the work unit. | ❏ | ❏ | ❏ | ❏ | ❏ | ❏ |
| People feel understood and accepted by each other. | ❏ | ❏ | ❏ | ❏ | ❏ | ❏ |
| Members of the work unit build on each other's ideas in order to achieve the best possible outcome. | ❏ | ❏ | ❏ | ❏ | ❏ | ❏ |
| People in the work unit cooperate in order to help develop and apply new ideas. | ❏ | ❏ | ❏ | ❏ | ❏ | ❏ |
| We can trust our supervisor. | ❏ | ❏ | ❏ | ❏ | ❏ | ❏ |

Have you ever participated in an online or offline course to improve your chances of employment?

1. Yes
2. No

Where did you receive the training or courses?

1. Local University
2. Non-local University through distance learning
3. Massively open online courses, MOOCs such as Coursera, Udemy
4. Library
5. At a community center
6. At a high school
7. At some other place I haven’t mentioned

Did the training or classes help you (You can choose your top THREE reasons)

1. Expand my professional networks
2. Enable you to find a new job, whether that’s with your current organization or a different one.
3. Help you consider a different career path
4. Help you advance within your current company or organization
5. Opened up new perspectives about your life
6. Helped you make new friends
7. Made you feel more connected to your local community
8. Helped you feel more capable or well-rounded
9. Gotten you more involved in volunteer opportunities

Were there any barriers to attending the courses (check all that apply)

1. Cost
2. Hearing
3. Language
4. Physical mobility
5. Caring responsibility
6. Time
7. Transportation
8. Health
9. No barriers

In general, would you say your health is

1. Excellent
2. Very good
3. Good
4. Fair
5. Poor

The following questions are about activities you might do during a typical day. Does your health now limit you in these activities? If so, how much?

|  | Yes, limited a lot | Yes, limited a little | No, not limited at all |
| --- | --- | --- | --- |
| Vigorous activities (running, lifting heavy objects, doing strenuous sports) | ❏ | ❏ | ❏ |
| Moderate activities (moving a table, pushing a vacuum cleaner, bowling or playing golf) | ❏ | ❏ | ❏ |
| Lifting or carrying groceries | ❏ | ❏ | ❏ |
| Climbing several flights of stairs | ❏ | ❏ | ❏ |
| Climbing one flight of stairs | ❏ | ❏ | ❏ |
| Bending, keeling, or stooling | ❏ | ❏ | ❏ |
| Walking more than one kilometre | ❏ | ❏ | ❏ |
| Walking half a kilometre | ❏ | ❏ | ❏ |
| Waking 100 metre | ❏ | ❏ | ❏ |
| Bathing or dressing yourself | ❏ | ❏ | ❏ |

During the past 4 weeks, have you had any of the following problems with your work or other regular daily activities as a result of your physical health?

|  | Yes | No |
| --- | --- | --- |
| Cut down the amount of time you spent on work or other activities | ❏ | ❏ |
| Accomplished less than you would like | ❏ | ❏ |
| Were limited in the kind of work or other activities | ❏ | ❏ |
| Had difficulty performing the work or other activities (for example, it took extra effort) | ❏ | ❏ |

These questions are about how you feel and how things have been with you during the past 4 weeks. For each question, please give the one answer that comes closest to the way you have been feeling. How much of the time during the past 4 weeks:

|  | All of the time | Most of the time | A good bit of the time | A little of the time | None of the time |
| --- | --- | --- | --- | --- | --- |
| Did you feel full of life? | ❏ | ❏ | ❏ | ❏ | ❏ |
| Have you been a nervous person? | ❏ | ❏ | ❏ | ❏ | ❏ |
| Have you felt so down in the dumps that nothing could cheer you up? | ❏ | ❏ | ❏ | ❏ | ❏ |
| Have you felt calm and peaceful? | ❏ | ❏ | ❏ | ❏ | ❏ |
| Did you have a lot of energy? | ❏ | ❏ | ❏ | ❏ | ❏ |
| Have you felt down? | ❏ | ❏ | ❏ | ❏ | ❏ |
| Did you feel worn out? | ❏ | ❏ | ❏ | ❏ | ❏ |
| Have you been a happy person? | ❏ | ❏ | ❏ | ❏ | ❏ |
| Did you feel tired? | ❏ | ❏ | ❏ | ❏ | ❏ |

During the past 4 weeks, have you had any of the following problems with your work or other regular daily activities as a result of any emotional problems (such as feeling depressed or anxious)?

|  | Yes | No |
| --- | --- | --- |
| Cut down the amount of time you spent on work or other activities | ❏ | ❏ |
| Accomplished less than you would like | ❏ | ❏ |
| Didn't do work or other activities as carefully as usual | ❏ | ❏ |

In general, how often do you get together socially with friends or relatives not living with you?

1. Everyday
2. Several times a week
3. About once a week
4. 2 or 3 times a month
5. About once a month
6. Once or twice every 3 months
7. Less often than once every 3 months

During the past 4 weeks, how much of the time has your physical health or emotional problems interfered with your social activities (like visiting friends, relatives, etc.)?

1. All of the time
2. Most of the time
3. Some of the time
4. A little of the time
5. None of the time

How often do you feel rushed or pressed for time?

1. Never
2. Once in a while
3. About half the time
4. Most of the time
5. Always

In general, how often do you participate in moderate or intensive physical activity for at least 30 minutes? (Moderate level physical activity will cause a slight increase in breathing and heart rate, such as brisk walking).

1. Not at all
2. Less than once a week
3. 1 to 2 times a week
4. 3 times a week
5. More than 3 times a week (but not everyday)
6. Everyday

How satisfied are you in below areas of your life?

|  | 1 | 2 | 3 | 4 | 5 | 6 | 7 |
| --- | --- | --- | --- | --- | --- | --- | --- |
| Your employment opportunities in your area? | ❏ | ❏ | ❏ | ❏ | ❏ | ❏ | ❏ |
| You employment opportunities in your industry? | ❏ | ❏ | ❏ | ❏ | ❏ | ❏ | ❏ |
| Your employment opportunities with secure jobs? | ❏ | ❏ | ❏ | ❏ | ❏ | ❏ | ❏ |
| Your employment opportunities with appropriately paid jobs? | ❏ | ❏ | ❏ | ❏ | ❏ | ❏ | ❏ |
| Your employment opportunities with flexible jobs? | ❏ | ❏ | ❏ | ❏ | ❏ | ❏ | ❏ |
| Your job-seeking channels? | ❏ | ❏ | ❏ | ❏ | ❏ | ❏ | ❏ |
| The amount of free time you have? | ❏ | ❏ | ❏ | ❏ | ❏ | ❏ | ❏ |
| In how you use your time? | ❏ | ❏ | ❏ | ❏ | ❏ | ❏ | ❏ |

Given your current needs and financial responsibilities, would you say that you and your family are...

1. Prosperous
2. Very comfortable
3. Reasonably comfortable
4. Just getting along
5. Poor
6. Very poor

Since January 2023, did any of the following happen to you because of shortage of money?

|  | Yes | No |
| --- | --- | --- |
| Could not pay electricity, gas or telephone bills on time | ❏ | ❏ |
| Could not pay the mortgage or rent on time | ❏ | ❏ |
| Pawned or sold something | ❏ | ❏ |
| Went without meals | ❏ | ❏ |
| Was unable to heat home | ❏ | ❏ |
| Asked for financial help from friends or family | ❏ | ❏ |
| Asked for help from welfare/community organisations | ❏ | ❏ |

When you are exposed to climate impacts such as extreme weather at work, have you experienced any of the following physical effects? Check all that apply

1. Dehydration
2. Breathing difficulties (e.g. due to smoke exposure)
3. Increased incidence of workplace injury
4. Reduction in sleep quality after work
5. Tiredness/fatigue
6. Reduced productivity due to extreme weather events
7. Excessive sweating
8. Headaches
9. Poor coordination/slower reaction time
10. Dizziness and/or nausea
11. Sunburn
12. Other increased bodily discomfort

Has exposure to climate impacts such as extreme weather resulted in any of the following changes to your work or job-seeking situation? Check all that apply.

1. Reduction in work hours
2. Loss of job
3. Giving up applying for a job that requires a long commute.
4. Giving up applying for a job that has poor considerations for my climate needs
5. Transport to/from/for work disrupted (e.g. by heat, extreme weather and disasters such as bushfires and floods)
6. Having to work additional hours due to emergency situations and natural hazards
7. Having to work that is outside of your job description due to an emergency situation
8. Inability to meet required workloads and targets
9. Loss of personal leave due to using it up during extreme weather events
10. Other

This question seeks to find out your power to adapt or mitigate climate impact. Please move the pointer to indicate how true the sentences are.

|  |  |
| --- | --- |
| I can decline any jobs with climate change impacts that has adverse impact on my health and well-being | ❏ |
| I have knowledge of the measures to mitigate the climate change impact at work. | ❏ |
| I have adapted these mitigating measures at work previously. | ❏ |

This question is asking you if you are or have been exposed to below mentioned emission sources. Please indicate the likelihood of the exposure by ticking the most appropriate choice for each source.

|  | No exposure | Little exposure | Somewhat exposed | Frequently exposed | Always exposed |
| --- | --- | --- | --- | --- | --- |
| Fuel combustion, chemical utilization, and waste disposal | ❏ | ❏ | ❏ | ❏ | ❏ |
| Production of crops or livestock | ❏ | ❏ | ❏ | ❏ | ❏ |
| Coal for electric power production | ❏ | ❏ | ❏ | ❏ | ❏ |
| Locomotives, commercial boars, aircraft ground operations, shipping | ❏ | ❏ | ❏ | ❏ | ❏ |
| Bush fires and hazard reduction burning | ❏ | ❏ | ❏ | ❏ | ❏ |
| Highway-capable passenger and commercial vehicles | ❏ | ❏ | ❏ | ❏ | ❏ |
| Other off-highway transportation | ❏ | ❏ | ❏ | ❏ | ❏ |
| Commercial cooking | ❏ | ❏ | ❏ | ❏ | ❏ |
| Road, residential, and other construction | ❏ | ❏ | ❏ | ❏ | ❏ |
| Home combustion of wood | ❏ | ❏ | ❏ | ❏ | ❏ |
| Dust from paved and unpaved roads | ❏ | ❏ | ❏ | ❏ | ❏ |
| Lawn/gardening, charcoal grilling | ❏ | ❏ | ❏ | ❏ | ❏ |

What best describes your gender?

1. Male
2. Female
3. Non-binary
4. Prefer not to answer

Are you of Aboriginal or Torres Strait Islander origin?

1. Yes
2. No
3. Prefer not to answer

What is the highest level of education you have completed?

1. Year 9 or below
2. Year 10 or equivalent
3. Year 12 or equivalent Certificate or Diploma
4. Undergraduate degree
5. Postgraduate degree
6. Other (please specify)

What is your citizenship status?

1. Citizen (born in Australia)
2. Citizen (by conferral)
3. Permanent resident
4. Temporary resident
5. Refugee
6. Asylum seeker
7. Stateless person

How would you describe your ethnicity?

1. Australian
2. Oceanian
3. North-west European
4. Southern and Eastern European
5. North African and Middle Eastern
6. South-East Asian
7. North-East Asian
8. Southern and Central Asian
9. Peoples of the Americas
10. Sub-Saharan African
11. Other

Can you tell me your country of birth?

1. Australia
2. Other English speaking country
3. Non-English speaking country

Which state do you live in?

1. NSW
2. VIC
3. QLD
4. SA
5. WA
6. TAS
7. ACT
8. NT

Which area do you live in?

1. Urban area
2. Regional area
3. Rural area
4. Remote area

Can you provide your marital status?

1. Married
2. Living with partner
3. Widowed
4. Divorced
5. Separated
6. Never married

These are the questions on your household members whom you are currently living together:

|  | Living together |
| --- | --- |
| What is the total number of household members? | 0  1  2  3  4  5  6  7  8  9 |
| How many children under 15? | 0  1  2  3  4  5  6  7  8  9 |
| How many children over 15? | 0  1  2  3  4  5  6  7  8  9 |
| What is the number of adults working? | 0  1  2  3  4  5  6  7  8  9 |
| What is the number of dependents? | 0  1  2  3  4  5  6  7  8  9 |

What is your FORTNIGHTLY income after tax ?

|  |
| --- |

What is your household's total FORTNIGHTLY income after tax ?

|  |
| --- |

Which of the following best describes your housing situation?

1. I own my home outright
2. I own my home with a mortgage
3. I own my home with a reverse mortgage
4. I am renting
5. I live in my accommodation rent-free
6. I live in residential aged care
7. Other

Thank you for completing this survey.
